# Predicting neoadjuvant chemotherapy treatment response in hormone-receptor-positive/HER2-negative breast cancer – results from the Swedish SCAN-B population-based cohort

**DOI:** 10.2340/1651-226X.2025.44201

**Published:** 2025-11-19

**Authors:** Niklas Loman, Hani Saghir, Siker Kimbung

**Affiliations:** a Division of Oncology, Department of Clinical Sciences, Lund University, Lund, Sweden; b Department of Hematology, Oncology and Radiation Physics, Lund University Hospital, Malmö/Lund, Sweden; c Lund University Cancer Center, Lund, Sweden; d Department of Hematology, Sahlgrenska University Hospital, Gothenburg, Sweden

**Keywords:** Neoadjuvant chemotherapy, hormone-receptor-positive, optimal response, endocrine responsiveness, personalized treatment

## Abstract

**Background and purpose:**

Hormone-receptor-positive/HER2-negative (HR+/HER2-) early-stage breast cancers (EBCs) display heterogenous responses to neoadjuvant chemotherapy (NACT) warranting biomarkers to tailor optimal treatment for individual patients.

**Patients/material and methods:**

Women with HR+/HER2- EBC (*N* = 178) included in the Swedish Sweden Cancerome Analysis Network-Breast (SCAN-B) population-based cohort (2010–2019) treated with NACT were included. We analyzed rates of pathologic complete response (pCR), objective response (OR), breast conserving surgery (BCS), and recurrence-free interval (RFI) in subgroups defined by baseline clinicopathological and molecular characteristics.

**Results:**

The pCR rate was low (6%); nonetheless, after a median follow-up of 5.41 years, all patients who achieved pCR remained recurrence-free despite uniform baseline predicted high PAM50 risk of recurrence (ROR). Younger age (≤ 40 years), cT1, ER% positivity (≤ 66%), and negative PR (≤ 10%) were conventional clinicopathological factors positively associated with increased pCR. Molecular predictors of pCR included negative HR status by gene-expression signatures and non-luminal PAM50 subtypes. Tumor shrinkage ≥ 30%, i.e., OR and BCS, was achieved in 59% and 34%, respectively. No factor was significantly associated with ORR, whereas non-lobular histology and cT1 were positively associated with BCS. In addition, only 1/49 patients who underwent BCS experienced a recurrence during follow-up. Low/intermediate ER% positivity, PR negativity, and non-luminal PAM50 subtype were baseline factors univariately prognostic for inferior long-term outcome in case of residual disease.

**Interpretation:**

Baseline characteristics indicative of reduced hormonal signaling and non-luminal tumor biology assessed more precisely using mRNA profiling can guide optimal tailoring of NACT for patients with high-risk HR+/HER2-tumors. Baseline molecular biology did not predict surgical outcomes following NACT.

## Introduction

Hormone-receptor-positive (HR+) and human epidermal growth factor receptor 2-negative (HER2-) breast cancer (BC) constitute ~70% of all breast malignancies and display substantial pathological and molecular heterogeneity, which impact treatment response and prognosis [1–3]. HR+/HER2- BC is often detected as a surgically resectable tumor allowing for upfront surgery followed by molecularly driven adjuvant treatment choices, as accessible. For patients presenting with larger tumors, a substantial lymph nodal burden, or those who may favor the less radical breast conservating surgery (BCS) as opposed to mastectomy, neoadjuvant therapy may be offered [4]. The decision to provide neoadjuvant chemotherapy (NACT) for patients with HR+/ HER2-tumors is still based on conventional clinical and pathological features rather than molecular/genomic biomarkers, in stark contrasts with the adjuvant setting where various gene-expression signatures have been widely adopted to support decision-making about chemotherapy use [5–7]. Without robust molecular biomarker-driven strategies, treatment selection based solely on the tumor staging is limited by the risk for exposing and/or overtreating patients with highly toxic drugs without guarantees of a meaningful clinical benefit.

Multiple randomized clinical trials have demonstrated that administering chemotherapy before surgery does not compromise long-term survival [8, 9] in spite of delaying the time to removal of the tumor, expanding clinical adoption of NACT. Moreover, providing chemotherapy treatment preoperatively allows for *in vivo* assessment of treatment response, opening avenues for tailoring adjuvant treatment according to response [4]. Pathologic complete response (pCR) following NACT is a robust prognostic biomarker for excellent long-term outcomes at the individual patient level, but pCR rates are substantially low (~5–15%) among patients with HR+/HER2- BC [10, 11]. Nonetheless, adjuvant endocrine therapy significantly improves long-term prognosis for patients with HR+/HER2- BC [12–14].

Although achieving a pCR is most desirable, the possibility for surgical down-staging remains a key outcome after NACT, especially in patients with large HR+/HER2- early-stage breast cancer (EBC). BCS rates up to 50% have been reported after NACT for such patients [15–17], indicating many patients do not achieve this clinical benefit. Response predictive markers are therefore needed to optimize NACT usage in this diverse clinical subtype of BC. Herein, we explore clinical, pathological, and molecular features associated with pCR, objective response (OR), BCS, and prognosis after NACT among patients with HR+/HER2- early BC included in the Sweden Cancerome Analysis Network-Breast (SCAN-B) cohort between 2010 and 2019.

## Patients/material and methods

### Patients

**Figure 1:**
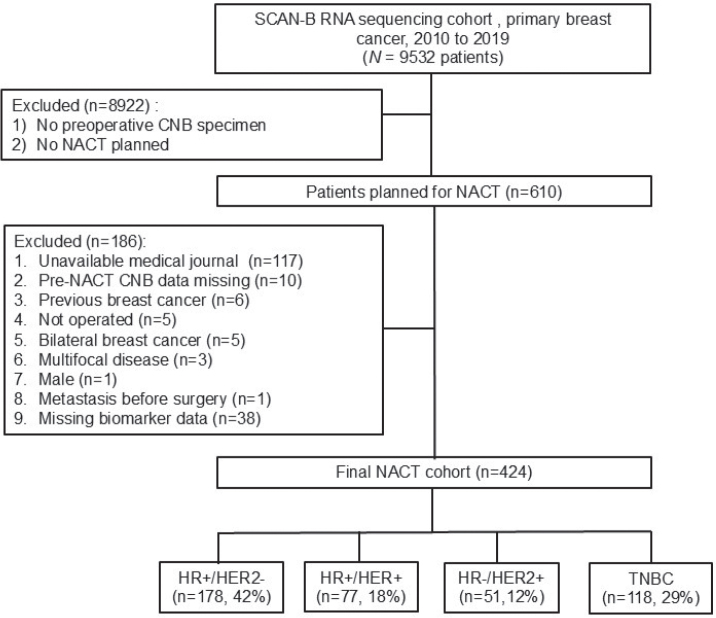
Consort diagram showing patient and tumor selection for inclusion in the analyses reported in the current study.

SCAN-B comprises a population-based consecutively enrolled series of BC patients accrued at nine hospitals in the Skåne, Uppland, and Småland healthcare regions in Sweden for the purpose of analyzing breast tumors using whole-genome RNA sequencing (RNAseq) technology [18–20]. The SCAN-B research database (inclusion period 2010–2019) was reviewed to identify women treated with NACT. The availability of relevant biomarker information assessed on the preoperative core needle biopsy (CNB) was necessary. Refer to the consort diagram (Figure 1) for details about subcohort selection for the current analyses.

### Patient and tumor characteristics

Information about patient demographics, conventional tumor histo-pathology characteristics, medical treatments, and pathological and surgical outcome after NACT were retrieved from the Swedish national quality register for BC (NKBC) [21] and complemented with review of patient medical records wherever possible. Preoperative anthracycline- and taxane-based chemotherapy regimens followed by postoperative endocrine therapy were the standard treatment regimen for patients with locally advanced inoperable HR+/HER2- tumors during the inclusion period. Neither neo-adjuvant endocrine treatment (NET), post-NACT nor adjuvant CDK 4/6 inhibitor was the standard treatment in Sweden between 2010 and 2019. Central reevaluation of biomarkers was not performed; hence, our results capture real-world experiences. Biomarkers were assessed on pre-treatment core biopsies only. Protocols and cutoffs for routine evaluation of tumor histology and hallmark biomarkers (ER, PR, HER2, and Ki67) status are described in guidelines published by the Swedish society of pathologists (KVAST) [22]. To explore the diverse expression of ER between tumors, we split the percentage ER positivity into three categories: low (≤ 10%), intermediate (11%–66%), and high (> 66%). The threshold for the low ER group was set at the ≤ 10% to reflect the recognized differential biology of ‘ER-low; 1–10%’ tumors [23–25]. HR+ tumors with low/intermediate ER positivity not only are more likely to be sensitive to NACT [25] but also represent a group where additional measures are needed in case of residual cancer at surgery. A threshold of 20% immunohistochemical staining was applied in the material description to discriminate between high vs. low proliferative disease. Although imperfect, this is a threshold generally utilized in clinical practice, e.g., in the monarchE study to identify patients eligible for the addition of adjuvant CDK 4/6 inhibitor treatment [26].

### RNA sequencing-derived hallmark features

The description of SCAN-B procedures for tissue collection, RNA sequencing, data cleaning, and the development of RNAseq-derived single sample predictor (SSP) models for annotating ER, PR, and Ki67 status and the PAM50 molecular subtypes and PAM50 genomic risk of recurrence (ROR) scores and risk category class have been published [18–20].

### Statistical analysis

Analyses in this study are restricted to patients with HR+/HER2- tumors, unless otherwise stated. The association of clinical pathological and molecular factors with time independent outcomes (pCR, OR, and surgical procedure) was evaluated using Fisher’s exact tests and logistic regression models outputting odds ratios (ORs) and 95% confidence interval (CI). Kaplan-Meier plots and Log-rank tests were used to analyze associations of pathological and surgical outcomes with recurrence-free survival (RFI) as defined by the Standardized Definitions for Efficacy End Points (STEEP) criteria [27]. RFI was defined as the time from BC diagnosis until any recurrence (local, regional, or distant) with death being a censoring event. All statistical tests were two-sided, and *p*-values < 0.05 were considered significant. Analyses were performed using SPSS v29 (IBM Corporation, Armonk, NY, USA).

## Results

### Patient and tumor characteristics at baseline

In this SCAN-B cohort of *N* = 424 patients who received NACT, 178 (42%) patients presented with clinical HR+/HER2- tumors (Figure 1) and are the focus of this study. Using the Swedish immunohistochemical (IHC) cut-off (≤ 10%) for HR positivity, 80% versus 18% versus 2% were ER+/PR+ versus ER+/PR- versus ER-/PR+, respectively. The median age at diagnosis was 55 years (range 25–75 years), median radiological tumor size was 30 mm (range 9–120 mm), 87% had positive nodal involvement, 88% had clinical stage I&II disease (AJCC 8th edition), and 78% of tumors were invasive ductal carcinomas. Most (89%) showed high ER% positivity (> 66%) by IHC, and 82% were considered highly proliferative (Ki67 > 20%) (Table 1), reflecting common characteristics for selecting patients for preoperative chemotherapy. Similarly using RNAseq-derived SSPs, 91% were ER positive, 67% were PR-positive, 70% were highly proliferative (Ki67), 79% were assigned a PAM50 luminal subtype, and 81% were classified as genomic high-risk category by SSP-ROR (Table 1). Notably, all (100%) combined HR-negative cases by SSP were classified non-luminal by PAM50, and most (75%) had a predicted high genomic ROR. Prevalent clinicopathological characteristics among patients with non-luminal tumors included older age (74%), larger tumors (81%), nodal positivity (84%), high proliferation (91%), and invasive ductal histology (79%).

**Table 1:** Demographics and tumor characteristics at screening of SCAN-B patients (2010–2019) undergoing neoadjuvant chemotherapy treatment for early breast cancer by clinical subtypes.

Factor	HR+/HER2- (*n* = 178, 42%)	HR+/HER2+ (*n* = 77, 18.2%)	HR-/HER2+ (*n* = 51, 12%)	TNBC (*n* = 118, 27.8%)
**Age**				
Median (range), years	55 (25–75)	55 (30–80)	60 (35–75)	52.5 (30–80)
≤ 40 years	30 (16.9%)	25 (32.5%)	7 (13.7%)	25 (21.2%)
> 40 years	148 (83.1%)	52 (67.5%)	44 (86.3%)	93 (78.8%)
Missing	0	0	0	0
**Clinical Stage**				
I	7 (4.1%)	5 (6.6%)	2 (4.1%)	8 (7.1%)
II	145 (84.3%)	63 (82.9%)	44 (89.8%)	95 (84.8%)
III	20 (11.6%)	8 (10.5%)	3 (6.1%)	9 (8%)
Missing	6	1	2	6
Tumor size*				
Median (range), mm	30 (9–120)	28.5 (10–90)	26 (11–100)	27 (12–120)
≤ 20 mm	37 (24.2%)	15 (22.4%)	13 (25.5%)	23 (20%)
> 20 mm	116 (75.8%)	52 (77.6%)	38 (74.5%)	92 (80%)
Missing	25	10	0	3
**Nodal Status**				
Negative	20 (12.9)	18 (26.5%)	14 (27.5%)	57 (51%)
Positive	155 (87.1%)	50 (73.5%)	37 (72.5%)	55 (49%)
Missing	23	9	0	6
**Histology**				
Ductal	137 (78.3%)	67 (89.3%)	43 (89.6%)	93 (80.9%)
Lobular	26 (14.9%)	5 (6.7%)	1 (2.1%)	3 (2.6%)
Mixed Ductal/lobular	1 (0.6%)	0 (0%)	0 (0%)	0 (0%)
Other invasive	11 (6.3%)	3 (4%)	4 (8.3%)	19 (16.5%)
Missing	3	2	3	3
**PR Status IHC**				
Negative (≤ 10%)	32 (18%)	20 (26%)	51 (100%)	118 (100%)
Positive (> 10%)	145 (82%)	57 (74%)	0 (0%)	0 (0%)
Missing	1	0	0	0
**ER% IHC**				
Median (range), %	99 (0–100)	95 (1–100)	0 (0–4)	0 (0–9)
< 10%	3 (1.7%)	1 (1.3%)	51 (100%)	118 (100%)
10–66%	16 (9%)	11 (14.3%)	0 (0%)	0 (0%)
> 66%	159 (89.3%)	65 (84.4%)	0 (0%)	0 (0%)
Missing	0	0	0	0
**ER/PR Status IHC**				
ER+/PR+	142 (80.3%)	56 (72.7%)	0(0%)	0 (0%)
ER+/PR-	32 (18%)	20 (26%)	0(0%)	0 (0%)
ER-/PR+	3 (1.7%)	1 (1.3%)	0%	0 (0%)
ER-/PR-	0%	0%	51 (100%)	118 (100%)
Missing	1	0	0	0
**Ki67% IHC**				
Median (range) %	33.5 (5–90)	39 (9–97)	45 (10–90)	60 (5–100)
≤ 20%	31 (17.8%)	4 (5.3%)	2 (3.9%)	5 (4.3%)
> 20%	143 (82.2%)	71 (94.7%)	49 (96.1%)	110 (95.7%)
Missing	4	2	0	3
**ER Status SSP**				
Negative	15 (9%)	16 (21%)	48 (98%)	113 (98%)
Positive	154 (91%)	61 (79%)	1 (2%)	2 (2%)
Missing	9	0	2	3
**PR Status SSP**				
Negative	55 (33%)	28 (36%)	47 (96%)	106 (92%)
Positive	114 (67%)	49 (64%)	2 (4%)	9 (8%)
Missing	9	0	2	3
**ER/PR Status SSP**				
ER+/PR+	114 (67%)	44 (57%)	1 (2%)	2 (2%)
ER+/PR-	40 (24%)	17 (22%)	0 (0%)	0 (0%)
ER-/PR+	0 (0%)	5 (7%)	1 (2%)	7 (6%)
ER-/PR-	15 (10%)	11 (14%)	47 (96%)	106 (92%)
Missing	9	0	2	3
**Ki67 SSP**				
Low	50 (30%)	9 (12%)	7 (14%)	7 (6%)
High	119 (70%)	68 (88%)	42 (86%)	108 (94%)
Missing	9	0	2	3
**PAM50 Subtype®**				
LumA	60 (35.5%)	10 (13%)	0 (0%)	2 (1.7%)
LumB	74 (43.8%)	21 (27.3%)	0 (0%)	1 (0.9%)
HER2-enriched	18 (10.7%)	43 (55.8%)	37 (75.5%)	16 (13.9%)
Basal-like	17 (10.1%)	3 (3.9%)	12 (24.5%)	96 (83.5%)
Missing	9	0	2	3
**PAM50-ROR Score**				
Median (Range)	70 (5–95)	75 (5–95)	90 (5–95)	95 (5–95)
Low	14 (10%)	3 (5%)	4 (10%)	8 (7%)
Intermediate	13 (9%)	2 (3%)	0 (0%)	5 (5%)
High	117 (81%)	60 (92%)	36 (90%)	94 (88%)
Missing	34	12	11	11

Tumor size*; Longest diameter of breast mass by Ultrasound.

PAM50 Subtype^®^; PAM50 subtypes akin to Prosigna subtypes.

Abbreviations: HR: hormone receptor; TNBC: triple-negative breast cancer; ER: estrogen receptor; PR: progesterone receptor; SSP: single sample predictor.

### Concordance between IHC and molecular biomarkers

The agreement between the IHC versus SSP status for ER and PR was 93% (kappa 0.33) and 79% (kappa 0.44), respectively. Discordant cases mainly switched from IHC-positive to SSP-negative; 100% and 83% for ER and PR status, respectively. Most IHC low/intermediate (≤ 66%) ER% positive and PR-negative tumors were non-luminal by PAM50 and had high genomic ROR (Supplementary Figure 1).

### Association of pCR with conventional clinicopathological characteristics among patients with clinical HR+/HER2– tumors

**Figure 2:**
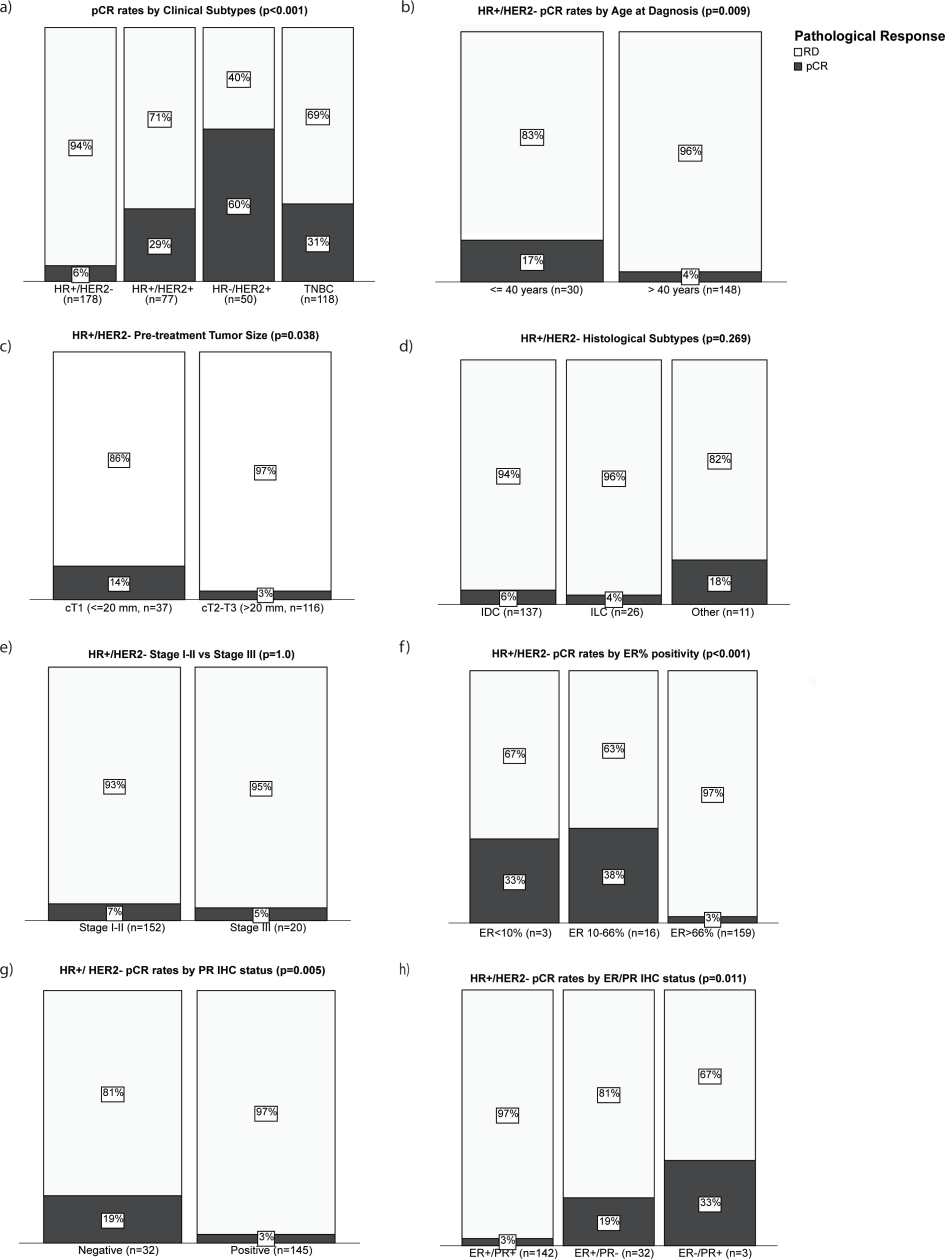
pCR rates by conventional clinicopathological features. Bar plots showing percentage of patients who achieved pCR (black) versus patients with residual cancer (white) by (a) clinical subtypes in all SCAN-B NACT cohort (*n* = 424), (b) age at diagnosis in HR+/HER2- disease, (c) pre-treatment tumor size in HR+/HER2- disease, (d) tumor histological subtypes in HR+/HER2- disease, (e) clinical disease stage in HR+/HER2- disease, (f) ER% positivity by IHC in HR+/HER2- disease, (g) PR status by IHC in HR+/HER2- disease, and (h) combined ER/PR status in HR+/HER2- disease. *P*-values are from Fisher’s exact 2-sided tests. Abbreviations: RD: residual disease; pCR: pathological complete response; IHC: immunohistochemistry.

pCR was defined as the disappearance of all signs of invasive cancer in the breast and axillary nodes (ypT0 ypN0). Only 6% (11/178) of patients with clinical HR+/HER- BC achieved a pCR (Figure 2a). Younger age at diagnosis (≤ 40 years) was associated with higher odds for pCR (OR = 4.7, 95% CI 1.3–16.7, Figure 2b). Age cut-off of 40 years is supported by results from the SOFT/TEXT trials, suggesting that patients with younger age have more aggressive tumors and worse 12-year overall survival [28, 29]. Patients with cT1 tumors were more likely to achieve a pCR compared to cT2-T3 tumors (13% vs. 9%, *p* = 0.038, Figure 2c), but histological subtype (*p* = 0.269; Figure 2d) and clinical stage (*p* = 1.0, Figure 2e) did not impact the rate of pCR. To further evaluate the pathological response to NACT, we explored the objective response rate (ORR) in relation to baseline characteristics. OR was calculated as > 30% percent reduction in tumor size when comparing preoperative ultrasound to the pathological size of the residual cancer (pre-surgical radiological size was not available), to mimic the RECIST 1.1 criteria for treatment response [30]. Optimally, the same imaging modality should be used to determine ORR. ORR for the clinical HR+/HER2- subtype was 59%; however, no baseline clinicopathological or molecular factor significantly associated with OR in our cohort (Table 2).

**Table 2:** Associations between the objective response rate and pre-treatment clinical and molecular tumor characteristics among patients with HR+/HER2- tumors.

Factor	Responders (≥ 30% shrinkage) (*n*, %)	Non-responders (< 30% shrinkage) (*n*, %)	*p*-value
**Age**			0.678
≤ 40 years	16 (55.2%)	13 (44.8%)	
> 40 years	75 (60%)	50 (40%)	
**Clinical Stage**			0.800
I&II	79 (59.8%)	53 (40.2%)	
III	10 (55.6%)	8 (44.4%)	
Tumor size*			0.341
≤ 20 mm	19 (51.4%)	18 (48.6%)	
> 20 mm	70 (60.9%)	45 (39.1%)	
**Histology**			0.204
Ductal	77 (61.6%)	48 (38.4%)	
Lobular	6 (37.5%)	10 (62.5%)	
Other invasive	6 (60%)	4 (40%)	
**PR Status IHC**			0.830
Negative	16 (61.5%)	10 (38.5%)	
Positive	75 (58.6%)	53 (41.4%)	
**ER% IHC**			0.310
≤ 66%	13 (72.2%)	5 (27.8%)	
> 66%	78 (57.4%)	58 (42.6%)	
**ER/PR Status IHC**			0.926
ER+/PR+	73 (58.4%)	52 (41.6%)	
ER+/PR-	16 (61.5%)	10 (38.5%)	
ER-/PR+	2 (66.7%)	1 (33.3%)	
Ki67% IHC*			0.584
≤ 20%	13 (14.4%)	11 (17.7%)	
> 20%	77 (85.6%)	51(82.3%)	
**ER Status SSP**			0.779
Negative	9 (64.3%)	5 (35.7%)	
Positive	79 (57.7%)	58 (42.3%)	
**PR Status SSP**			0.728
Negative	28 (56%)	22 (44%)	
Positive	60 (59.4%)	41 (40.6%)	
**ER/PR Status SSP**			0.682
ER+/PR+	60 (59.4%)	41 (40.6%)	
ER+/PR-	19 (52.8%)	17 (47.2%)	
ER-/PR-	9 (64.3%)	5 (35.7%)	
Ki67 SSP*			0.281
Negative	23 (51.1%)	22 (48.9%)	
Positive	65 (61.3%)	41 (38.7%)	
**PAM50 Subtype®**			0.734
Luminal A	32 (60.4%)	21 (39.6%)	
Luminal B	36 (54.5%)	30 (45.5%)	
Non-Luminal	20 (62.5%)	12 (37.5%)	
**PAM50-ROR Category**			0.509
Low/Intermediate	15 (55.6%)	12 (44.4%)	
High	70 (63.6%)	40 (36.4%)	

Tumor size*; Longest diameter of breast mass by Ultrasound.

HR: hormone receptor; ER: estrogen receptor; PR: progesterone receptor; SSP: single sample predictor.

The impact of the IHC quantitative HR% positivity on the pCR rate was next explored. Tumors with low (≤ 10%) and intermediate (11–66%) ER% positivity had higher pCR rates compared to high (> 66%) ER% positive tumors (33% vs. 38% vs. 3%, respectively; *p* < 0.001; Figure 2f). Similarly, pCR was more frequent among PR-negative (≤ 10%) compared to PR-positive (> 10%) tumors (19% vs. 3%, respectively; *p* = 0.005; Figure 2g). Furthermore, combined ER+/PR+ (> 10% cutoff for both receptors) was associated with the lowest pCR rate; 3% vs. 19% vs. 33% for ER+/PR+, ER+/PR-, and ER-/PR+, respectively (*p* = 0.011; Figure 2h).

### Association of pCR with baseline molecular biomarkers among patients with clinical HR+/HER2– tumors

**Figure 3:**
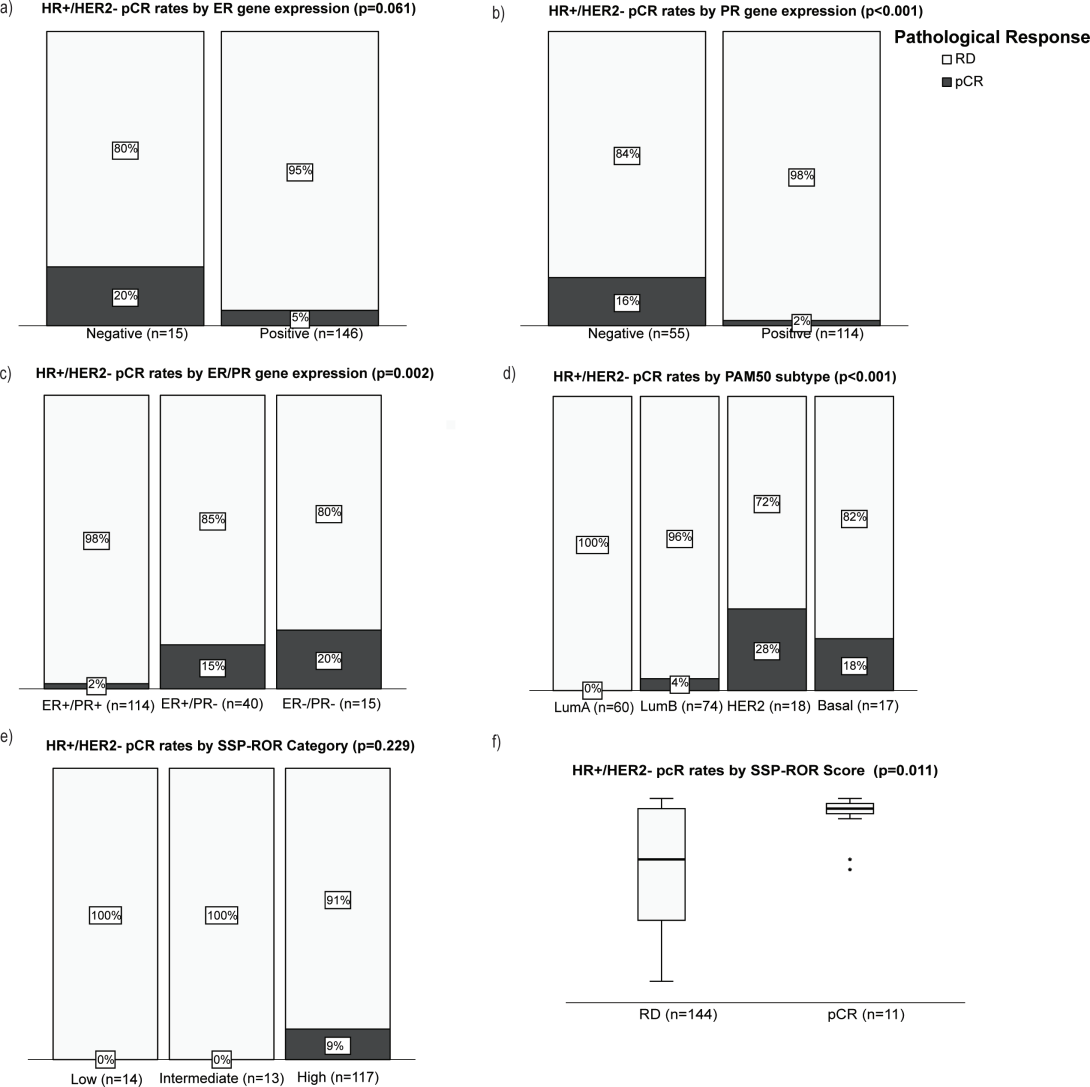
pCR rates by molecular (mRNA-based) biomarkers in HR+/HER2- disease. Bar plots showing percentages of patients who achieved pCR (black) versus patients with residual cancer (white) by (a) ER gene signature status, (b) PR gene signature status, (c) combined HR gene signature status, (d) PAM50 molecular subtype, and (e) genomic risk of recurrence (SSP-ROR) category. Continuous SSP-ROR scores between cases with pCR and residual disease (f). *P*-values are from Fisher’s exact 2-sided tests (a–e) and Mann-Whitney test (f).

The odds for achieving a pCR was higher among clinical HR+ tumors classified by SSP to be ER-negative (OR = 4.5, 95% CI 1.1–19.5, Figure 3a) or PR-negative (OR = 11, 95% CI 2.3–52.7, Figure 3b). Moreover, combined ER-/PR- by SSPs was associated with a higher pCR rate (20%) compared to ER+/PR- (15%) or ER+/PR+ (2%), respectively (*p* = 0.002; Figure 3c). Note that no tumor was classified as ER-/PR+ by SSPs. Rates of pCR also varied significantly by PAM50 subtypes as follows: HER2-enriched (28%) versus basal (18%) versus LumB (4%) versus LumA (0%) [*p* < 0.001; Figure 3d]. Precisely, 8/11 (72%) patients who achieved a pCR had a non-luminal tumor by PAM50 classification, and all (11/11, 100%) were predicted to have a high genomic ROR (Figure 3e and f).

### Multivariable logistic regression analyses

Logistic regression analyses adjusted pairwise for covariates (pCR ~ biomarker + covariate) were performed to further understand the association between pCR and the biomarkers age at diagnosis (≤ 40 years vs. > 40 years), IHC ER% positivity (≤ 66% vs. > 66%), and PAM50 subtype (non-luminal vs. luminal A/B); Supplementary Table 1. Covariates included pre-treatment tumor size (≤ 20 mm vs. > 20 mm), clinical stage (I&II vs. III), IHC PR status (positive vs. negative), and histology subtype. Low/intermediate ER% positivity (≤ 66%) remained independently associated with higher odds for pCR in all pairwise adjusted models. Younger age at diagnosis was significantly associated with higher odds for pCR in all pairwise models except when adjusting for PAM50 subtype (*p* = 0.057). Likewise, non-luminal PAM50 subtype was significantly associated with higher odds for pCR in all pairwise except when adjusting for ER% positivity (*p* = 0.271). In a multivariable model that combined age, tumor size, ER% positivity, and PR status by SSP and PAM50 subtype, only ER% positivity and tumor size emerged as independent positive predictive factors for pCR after NACT (*p* = 0.012. and *p* = 0.008, respectively). Note that the PAM50 ROR category was exempted from these exploratory logistic regression analyses because all pCR cases converged within the high-risk category, making odds-ratio calculation unreliable due to the statistical problem of ‘complete separation’.

### Association of surgical outcomes with baseline clinical and molecular factors and pathological response

The rate of BCS after NACT among patients with clinical HR+/HER2- subtypes was 34%. Smaller tumor size (cT1) and non-lobular histology were the only pathological factors positively associated with BCS over mastectomy in this cohort. Notably, neither baseline molecular characteristics nor ORR after NACT was associated with BCS versus mastectomy (Table 3).

**Table 3:** Associations of surgical outcomes with pre-treatment clinical and molecular factors and pathological response to NACT among patients with HR+/HER2- tumors.

Factor	BCS (*n*, %)	Mastectomy (*n*, %)	*P*-value
**Age**			0.166
≤ 40 years	5 (20%)	20 (80%)	
> 40 years	48 (36.1%)	85 (63.9%)	
**Clinical Stage**			0.115
I&II	50 (37%)	85 (63%)	
III	3 (16.7%)	15 (83.3%)	
Tumor size*			**0.01**
≤ 20 mm	17 (56.7%)	13 (43.3%)	
> 20 mm	31 (30.1%)	72 (69.9%)	
**Histology**			**0.007**
Ductal	47 (38.5%)	75 (61.5%)	
Lobular	1 (4.5%)	21 (95.5%)	
Other invasive	3 (30%)	7 (70%)	
**PR Status IHC**			0.380
Negative	7 (25%)	21 (75%)	
Positive	45 (34.9%)	84 (65.1%)	
**ER% IHC**			0.581
≤ 66%	4 (25%)	12 (75%)	
> 66%	49 (34.5%)	93 (65.5%)	
**ER/PR Status IHC**			0.572
ER+/PR+	44 (34.6%)	83 (65.4%)	
ER+/PR-	7 (25%)	21 (75%)	
ER-/PR+	1 (50%)	1 (50%)	
Ki67% IHC*			0.343
≤ 20%%	7 (13.5%)	20 (19.6%)	
> 20%	45 (86.5%)	82 (80.4%)	
**ER Status SSP**			1.0
Negative	5 (35.7%)	9 (64.3%)	
Positive	46 (33.8%)	90 (66.2%)	
**PR Status SSP**			0.468
Negative	15 (29.4%)	36 (70.6%)	
Positive	36 (36.4%)	63 (63.6%)	
**ER/PR Status SSP**			0.596
ER+/PR+	36 (36.4%)	63 (63.6%)	
ER+/PR-	10 (27%)	27 (73%)	
ER-/PR-	5 (35.7%)	9 (64.3%)	
Ki67 SSP*			0.851
Negative	14 (32.6%)	29 (67.4%)	
Positive	37 (34.6%)	70 (65.4%)	
**PAM50 Subtype®**			0.195
Luminal A	15 (30.6%)	34 (69.4%)	
Luminal B	28 (41.2%)	40 (58.8%)	
Non-Luminal	8 (24.2%)	25 (75.8%)	
**PAM50-ROR Category**			0.333
Low/Intermediate	10 (47.6%)	11 (52.4%)	
High	38 (35.8%)	68 (64.2%)	
**pCR**			0.497
Yes	2 (20%)	8 (80%)	
No	51 (34.5%)	97 (65.5%)	
**Objective Response**			0.464
< 30% tumor shrinkage	17 (31.5%)	37 (68.5%)	
≥ 30% tumor shrinkage	31 (38.8%)	49 (61.3%)	

Tumor size*; Longest diameter of breast mass by Ultrasound.

Abbreviations: BCS: breast conserving surgery; NACT: neoadjuvant chemotherapy; HR: hormone receptor; ER: estrogen receptor; PR: progesterone receptor; SSP: single sample predictor.

### Associations of recurrence-free interval (RFI) by baseline clinic-pathological and molecular biomarkers and post-NACT outcomes

**Figure 4:**
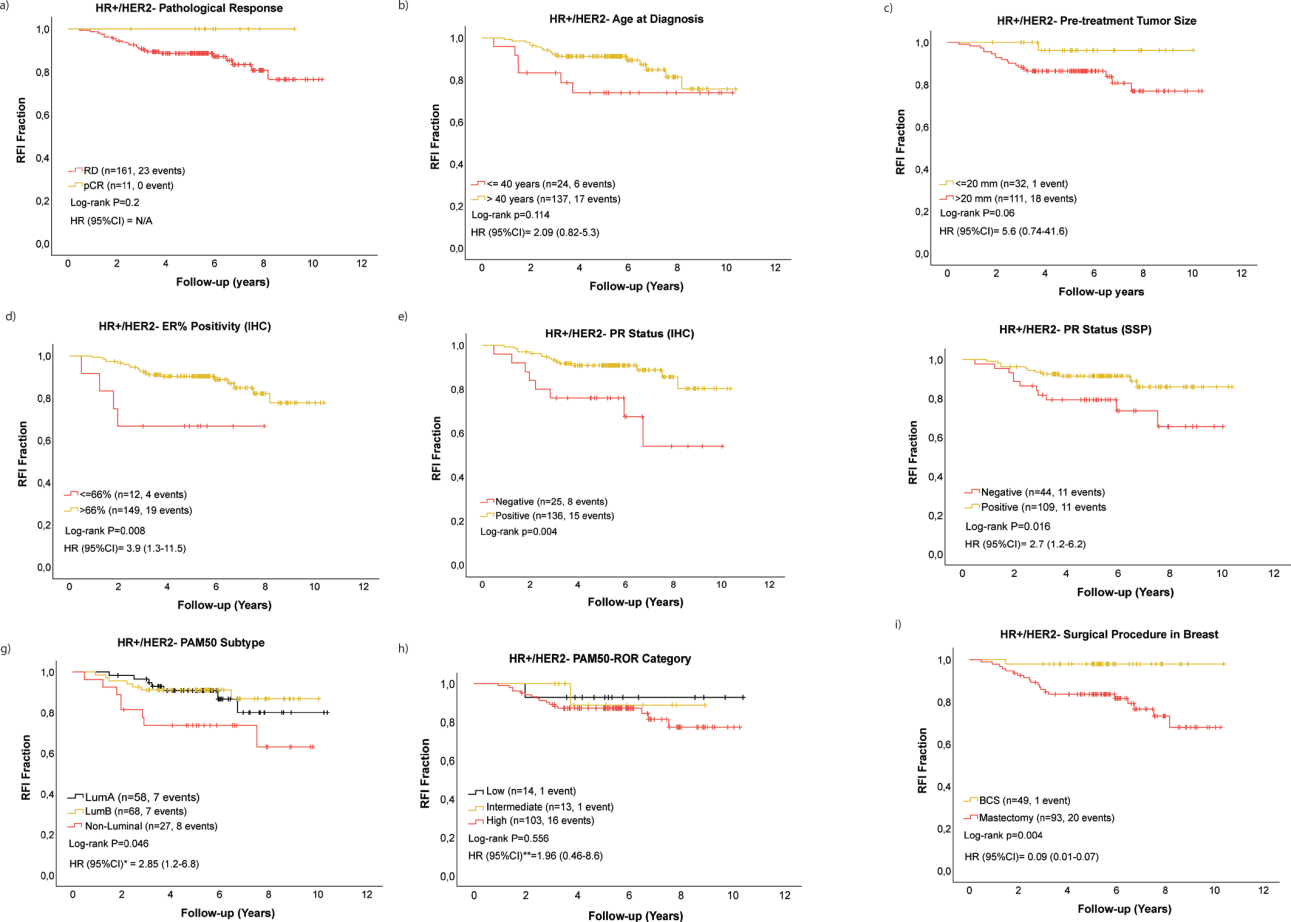
Recurrence-free interval (RFI) stratified by pCR status among all patients with HR+/HER2- tumors (a). RFI among patients with HR+/HER2- with residual disease stratified by age at diagnosis (b), pre-treatment tumor size (c), ER% positivity IHC (d), PR status IHC (e), PR status SSP (f), PAM50 subtype (g), PAM50-ROR category class (h), and breast surgery procedure (i).

RFI data were available for 172/178 patients with HR+/HER-, of which 24 registered an event during a median follow-up for RFI of 5.41 years (range 0.5–10.4 years). Despite the general high genomic ROR predicted at baseline, all patients who achieved a pCR remained disease-free during follow-up [HR = 0.05, 95% CI 0.00–59, Figure 4a]. Among patients with residual disease, younger age [≤ 40 years old; HR 2.1 (95% CI 0.82–5.3), Figure 4b] and larger tumor size [> 20 mm; HR = 5.6, 95% CI 0.74–41.6, Figure 4c] trended toward shorter RFI, while low/intermediate ER% positivity (≤ 66%) [HR = 3.9, 95% CI 1.3–11.5, Figure 4d], IHC PR negativity [HR = 3.3, 95% CI 1.4–7.8, Figure 4e], SSP PR-negative status (HR = 2.7, 95% CI 1.2–6.8, Figure 4f), and non-luminal PAM50 subtype [HR = 2.9, 95% CI 1.2–6.8, Figure 4g] were significant univariate predictors of inferior RFI. Likewise, patients with predicted high PAM50 ROR displayed a non-significant trend for worse prognosis relative to low/intermediate ROR categories (HR = 1.98, 95% CI 0.46–8.6, Figure 4h). Interestingly, BCS was associated with significantly fewer RFI events compared to mastectomy [HR = 0.09, 95% CI 0.01–0.7, Figure 4i]. Nevertheless, no factor was independently prognostic for RFI in multivariable analyses (Supplementary Table 2).

*represents HR (95% CI) for Non-luminal versus Lum A/B.

**represents HR (95% CI) for PAM50-ROR category class high versus low/intermediate.

N/A because hazard ratio and confidence intervals cannot be computed due to no pCR event in the pCR group.

## Discussion and conclusion

NACT is still used in the clinical management of early HR+/HER2-, despite the low likelihood for pCR, a less pronounced prognostic value, and the effect on adjuvant treatment. In this Swedish population-based SCAN-B cohort, over 40% of the women who received NACT as part of their oncological treatment between 2010 and 2019 presented with HR+/HER2- tumors, with only 6% achieving a pCR after NACT. The observed low pCR rate is consistent with previous studies [25, 31–33] and reinforces the necessity for optimal tailoring of chemotherapy treatment preoperatively in high-risk HR+/HER2- EBC. Standard clinicopathological biomarkers including younger age at diagnosis (≤ 40 years), cT1, low/intermediate ER% score (≤ 66%), and PR negativity (≤ 10%) univariately predicted higher pCR potential in our cohort. The association of cT1 with pCR (5 of 11 pCR cases were cT1) is surprising, but we noted that the responding cT1 tumors were biologically non-luminal (4/5 were non-luminal subtype). Huppert et al. [25] also found a positive association between pCR and low/intermediate ER% positivity (≤ 66), whereas another study including 10 neoadjuvant trials from the German Breast Group reported double pCR rates among ER+/PR- compared to ER+/PR+ tumors [33]. Claims of higher benefits of chemotherapy in premenopausal patients are however well documented in literature [6]. However, no factor was independently prognostic of pCR in our cohort, likely due to small subgroup sizes, which underpowered statistical analyses.

The biological and prognostic diversity within clinical HR+/HER2- EBC is well captured by gene-signatures and genomic prognostic scores, which are mostly clinically implemented in guiding adjuvant treatment decisions. The value of mRNA assays is not firmly established in preoperative treatment in HR+/HER2- EBC, where biomarkers for tailoring optimal treatment are needed. We explored the predictive and prognostic value of our SCAN-B developed RNAseq SSPs [19] for response to NACT in HR+/HER2- EBC. The HR SSPs assigned a ‘negative’ expression label to a substantial fraction of clinical HR+/HER2- tumors in our cohort, and one fifth was assigned a non-luminal PAM50 subtype, confirming that significant heterogeneity related to HR signaling persists within this clinical subgroup [7, 34]. Importantly, decreased hormonal signaling and non-luminal traits were predictive of higher pCR rates, underscoring the proven benefit of chemotherapy for typical endocrine non-responsive tumors [35]. In line with our results, Ohara et al. [36] reported a higher pCR rate among non-luminal PAM50 intrinsic subtypes while Whitworth et al. [34] and Huppert et al. [25] reported higher pCR rates for Blueprint-Basal-type subtype within clinical HR+/HER2- EBC. We observe that no LumA tumor in our series achieved a pCR, highlighting their intrinsic resistance to chemotherapy and projecting LumA subtype as a biomarker for non-pCR. Luminal A-like tumors (IHC-based) also presented with very low pCR rate in the large meta-analysis by Cortazar et al. [35]. Nonetheless, a few patients with low-grade luminal EBC still experience significant long-term benefits from (neo)adjuvant chemotherapy despite not achieving a pCR [37], thanks to adjuvant endocrine therapies.

Our SCAN-B developed PAM50 ROR score, and risk category class [19] assigned 81% of our cohort into the high-risk category at baseline. Notably, all patients with pCR were also predicted to have high genomic ROR. Though exploratory, our results align with previous studies reporting higher pCR rates among genomic high-risk categories assigned by signatures such as PAM50-ROR, OncotypeDX, Mammaprint, Blueprint, and EndoPredict [14, 25]. Larger and prospective studies are required to firmly establish the role of these molecular signatures in preoperative HR+/HER2- EBC. NET alone is nowadays recommended for strongly HR+ tumors, based on comorbidities or low-risk luminal biology based on clinical characteristics and/or genomic signatures, but NACT remains a therapeutic contender for a selected few. Adjuvant CDK 4/6-based treatment today is offered to patients with intermediate or high risk. Although the treatment may be well tolerated, it is obvious that it leads to a prolonged treatment period (an additional 2–3 years of treatment), and potentially complicating rehabilitation and thereby delaying reentry to the patients ‘healthy life’. Adjuvant CDK-inhibitor treatment also comes with a high cost both for the drug itself and most likely also associated with a prolonged period on sick-leave. Additional prognostic information acquired as a consequence of preoperative treatment potentially could be helpful in selecting patients who could potentially omit such additional treatment modalities.

Although an OR (≥ 30% tumor shrinkage) was achieved in 59% of HR+/HER2- tumors after NACT in our cohort, no baseline patient or tumor characteristic was significantly associated with tumor shrinkage besides the unexpected link between cT1 and pCR. Predicting the potential for BCS upfront NACT treatment remains a clinical challenge. The rate of BCS after NACT in our cohort was 34% similar to rates reported for HR+/HER2- tumors in the ACOSOG Z1701 Alliance trial from 2009 to 2011 [15]. Rates of BCS up to 50% were reported in another large cohort of HR+/HER2- tumors (2011–2016; *n* = 2,237), although this high BCS rate was associated with a higher rate of involved margins [16]. Interestingly, ORR was not associated with BCS. In fact, 80% of the patients who achieved a pCR had a mastectomy compared to 66% mastectomy rate among cases with residual disease. Small tumor size and non-lobular histology were the only baseline factors univariately associated with lumpectomy versus mastectomy in our cohort. A previous publication including a subset preoperative SCAN-B patients of all clinical subtypes (2014–2019) suggested that the strongest predictors of BCS after preoperative chemotherapy are clinical stage at diagnosis and a low mammographic density [17]. Lobular histology was also identified as an independent predictor of mastectomy but not pCR after NACT previously [38, 39] and represents the most established negative-predictive factor for achieving BCS after NACT. Importantly, the choice for BCS after NACT can be influenced by other clinical factors and might affect surgical preferences, such as occurrence of extensive microcalcifications, unfavorable breast/tumor ratio, surgeon’s experience and attitude, BRCA mutation carrier status, and non-clinical factors like patient preference, regardless of OR to NACT.

The validity of pCR for predicting favorable long-term prognosis among patients with HR+/HER2- EBC [35, 40, 41] was supported by our results. Despite uniform molecular predicted high ROR scores at baseline, no patient who achieved a pCR experienced disease recurrence after a median follow-up of 5.41 years, although statistically non-significant, limited by the small numbers of patients and events included in the analysis. We noted that BCS correlated with excellent RFI probably associated with the high adjuvant radiotherapy rate (95%) in our cohort, and the enrichment for smaller tumors within the BCS group. Prognostic biomarkers for inferior RFI among patients with residual disease included tumor characteristics suggestive of non-luminal biology and endocrine non-responsiveness. Younger-age, larger tumor size, and high PAM50 ROR category class showed trends for inferior outcome. The fact that the biomarkers predictive for pCR adversely impact prognosis when treatment did not result in pCR stresses the context-dependency when utilizing biomarkers. How residual disease biology impacts prognosis remains an unsettled research question. In the study, we identify a group of ER+/HER2- tumors with an intermediate ER-expression (IHC 10–66%) with a higher likelihood of pCR following NACT, but at higher ROR in case of residual invasive cancer at surgery. In this prognostically adverse subset, characterized by an over representation of a non-luminal subtype by with gene expression profiling, there is a clinical need to evaluate the optimal post-neoadjuvant therapeutic approach.

Understanding biological heterogeneity is key to tailoring treatment. Our study is limited by the relatively small number of cases, exemplifying the limited use of NACT within this sub-population. The median follow-up for adverse events was only 5.41 years, which is not sufficiently long for HR+/HER2- BC, as late events occurring after 10 years follow-up are typically expected. Furthermore, NET and postoperative CDK4/6 inhibitors are nowadays included in the arsenal of therapies for early HR+/HER2- BC with significant improvements in prognosis. Nottingham hostologic grade (NHG) was mostly performed post-operatively (only 9% at baseline) during the treatment period; hence, it was not adjusted for in our analyses. Technical variability associated with tissue collection methods, processing, and interpretation of IHC stains is other limitations when data are collected from multiple laboratories. Nevertheless, our study uniquely describes Swedish population-based real-world outcomes of a decade of anthracycline and taxane-based NACT in HR+/HER2- EBC, a subgroup where the use of NACT is somewhat controversial. Our results are mostly consistent with independent and larger international cohorts of similarly treated patients, indicating that optimal tailoring of NACT in high-risk HR+/HER2- EBC is feasible using conventional IHC biomarkers and more precisely gene-signatures that capture non-luminal biology within clinical HR+ BCs. Future investigations are warranted in larger and prospective cohorts of patients exposed to relevant therapies and where tumor biomarkers are centrally evaluated.

## Supplementary Material



## Data Availability

RNA-sequencing-based gene-expression data, clinicopathological data, and SSP classifications for SCAN-B cohort were made publicly available and are retrievable at https://github.com/StaafLab/sspbc. However, information and mechanisms for accessing the specific subset of gene-expression and clinical data associated with the findings in this study can be obtained from the corresponding author upon reasonable request.
