# Supplementary material for: Predicting neoadjuvant chemotherapy treatment response in hormone-receptor-positive/HER2-negative breast cancer – results from the Swedish SCAN-B population-based cohort
Source: Acta Oncol. 2025 Nov 19;64:44201. doi: 10.2340/1651-226X.2025.44201 (PMC12645220; doi:10.2340/1651-226X.2025.44201)

Supplementary material has been published as submitted. It has not been copyedited, or typeset by Acta Oncologica

Supplementary Table 1: pCR associations for age at diagnosis, IHC ER% positivity, and PAM50 subtype among patients with HR+/HER2- tumors. Multivariable models are adjusted pairwise for indicated covariates.

**Covariate**	**Age (≤40 yrs)**		**ER% positivity (≤66%%)**		**PAM50 (non-luminal)**		**PAM50-ROR Category (high)**	
	OR [95%CI]	p-value	OR [95%CI]	p-value	OR [95%CI]	p-value	OR [95%CI]	p-value
Univariable	4.7 (1.3-16.7)	**0.016**	22.6 (5.8-88.21)	**<0.001**	12.9 (3.2-52)	**<0.001**	N/A	N/A
Age (≤40 yr vs >40 yr)	N/A	N/A	22.5 (5.4-93.5)	**<0.001**	12 (2.9-49.1)	**<0.001**	N/A	N/A
Size (≤20mm vs >20mm)	6.3 (1.5-26.2)	**0.011**	65.5 (7.1-608.04)	**<0.001**	26.9 (4.4-166)	**<0.001**	N/A	N/A
clinical stage (I&II vs III)	4.7 (1.3-16.7)	**0.016**	27.4 (6.7-112.7)	**<0.001**	14.9 (3.6-61.3)	**<0.001**	N/A	N/A
Histology	4.7 (1.3-16.8)	**0.019**	22.2 (5.4-90.6)	**<0.001**	14.9 (3.5-63.7)	**<0.001**	N/A	N/A
ER% (≤66% vs >66%)	4.7 (1.1-20.6)	**0.040**	N/A	N/A	3.1 (0.41-23.3)	0.271	N/A	N/A
PR status IHC (pos vs neg)	4.9 (1.7-28.5)	**0.007**	15.6 (3.5-69.4)	**<0.001**	11.1 (2.7-46.4)	**<0.001**	N/A	N/A
Combined ER/PR status IHC	6.0 (1.4-25.7)	**0.016**	13.8 (2.7-70.8)	**0.002**	9.8 (2.3-42.6)	**0.002**	N/A	N/A
ER status SSP (pos vs neg)	3.9 (1.1-14.1)	**0.040**	49.4 (8.4-290.4)	**<0.001**	14.6 (3.2-67.1)	**<0.001**	N/A	N/A
PR status SSP (pos vs neg)	4.6 (1.2-17.9)	**0.027**	12.6 (2.5-63.3)	**0.002**	6.9 (1.6-30.4)	**0.011**	N/A	N/A
PAM50 **(**non-Lum vs Lum A/B)	3.8 (0.96-14.9)	0.057	11.1 (1.5-77.3)	**0.015**	N/A	N/A	N/A	N/A

N/A because odds ratios and confidence intervals cannot be computed. No pCR event was registered in the low/intermediate ROR category, hence it impossible to calculate odds-ratio due to the statistical problem of "complete separation"

Abbreviations: Confidence interval (CI); odds ratio (OR); Estrogen receptor (ER); Progesterone receptor (PR); Luminal (Lum); year (yr); versus (vs)

Supplementary Table 2: Multivariable Cox regression analyses for Recurrence-free interval among patients with HR+/HER2- tumors with residual disease after NACT.

	Univariable		Multivariable	
**Covariates**	**HR [95%CI]**	**P-value**	**HR [95%CI]**	**P-value**
Age (≤40 yr vs >40 yr)	2.09 [0.82-5.3]	0.122	2.23 [0.74-6.6]	0.154
Size continuous	1.02 [0.99-1.04]	0.104	1.02 [0.99-1.04]	0.119
ER% (≤66% vs >66%)	3.9 [1.3-11.5]	**0.015**	1.69 [0.27-10.7]	0.578
PR status IHC (pos vs neg)	3.3 [1.4-7.8]	**0.007**	3.02 [0.93-1.04]	0.067
PAM50 (non-Lum vs Lum A/B)	2.85 [1.2-6.8]	**0.018**	1.42 [0.38-5.3]	0.600
Surgery (BCS vs Mastectomy)	0.09 [0.013-0.701]	**0.021**	0.14 [0.02-1.14]	0.066

Abbreviations: Neoadjuvant chemotherapy (NACT); Confidence interval (CI); Hazards ratio (OR); Estrogen receptor (ER); Progesterone receptor (PR); Luminal (Lum); year (yr); versus (vs)

Supplementary Figure 1: Distribution of PAM50 subtype and genomic risk of recurrence (SSP-ROR) categories by ER% positivity by IHC (a and c, respectively) and combined ER/PR status IHC status(b and d, respectively). P-values are from Fisher's exact 2-sided tests.

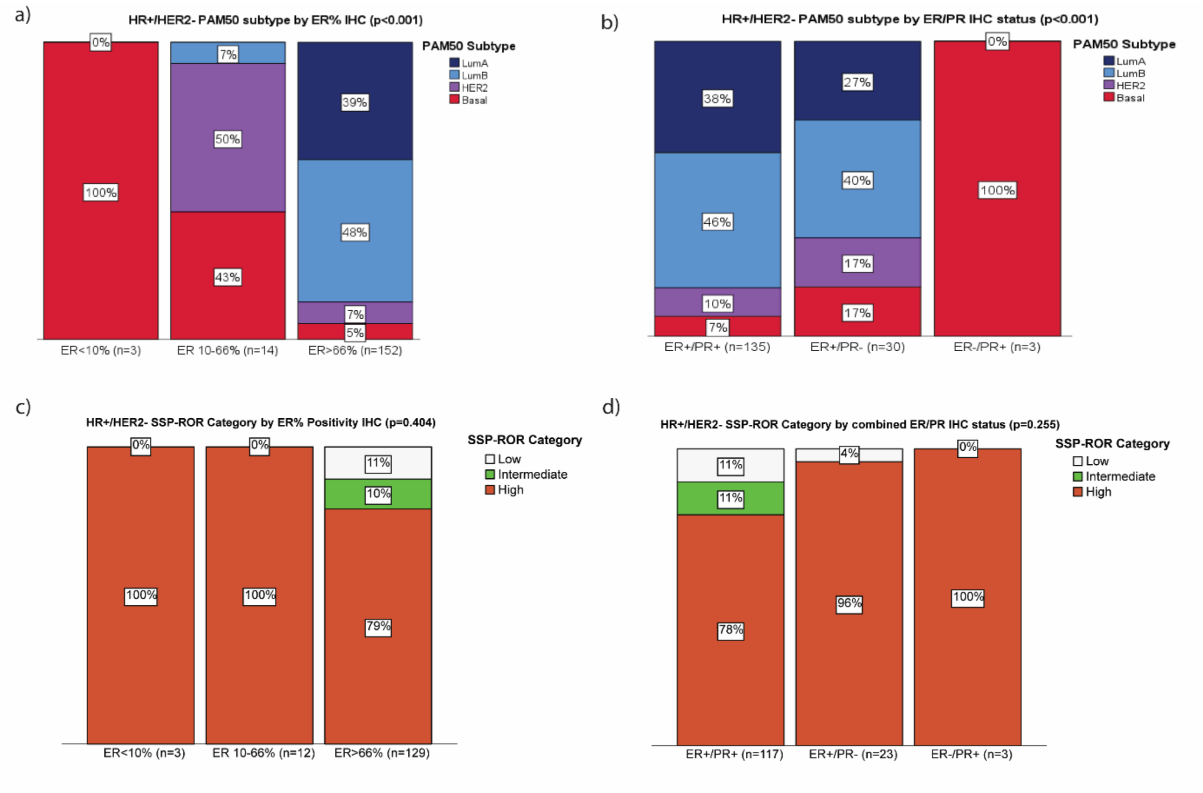

Supplement: Supplementary file 1 [file AO-64-44201-s1.pdf]
